# What do cancer-specific T cells ‘see’?

**DOI:** 10.1093/discim/kyac011

**Published:** 2022-12-06

**Authors:** Sabaria Shah, Abdullah Al-Omari, Katherine W Cook, Samantha J Paston, Lindy G Durrant, Victoria A Brentville

**Affiliations:** Scancell Limited, University of Nottingham Biodiscovery Institute, University Park, Nottingham, UK; Scancell Limited, University of Nottingham Biodiscovery Institute, University Park, Nottingham, UK; Scancell Limited, University of Nottingham Biodiscovery Institute, University Park, Nottingham, UK; Scancell Limited, University of Nottingham Biodiscovery Institute, University Park, Nottingham, UK; Scancell Limited, University of Nottingham Biodiscovery Institute, University Park, Nottingham, UK; Division of Cancer and Stem Cells, School of Medicine, University of Nottingham Biodiscovery Institute, University Park, Nottingham, UK; Scancell Limited, University of Nottingham Biodiscovery Institute, University Park, Nottingham, UK

**Keywords:** T cells, tumour antigen, mutational neo-epitope, post-translational modification, virus-associated tumour

## Abstract

Complex cellular interactions between the immune system and cancer can impact tumour development, growth, and progression. T cells play a key role in these interactions; however, the challenge for T cells is to recognize tumour antigens whilst minimizing cross-reactivity with antigens associated with healthy tissue. Some tumour cells, including those associated with viral infections, have clear, tumour-specific antigens that can be targeted by T cells. A high mutational burden can lead to increased numbers of mutational neoantigens that allow very specific immune responses to be generated but also allow escape variants to develop. Other cancer indications and those with low mutational burden are less easily distinguished from normal tissue. Recent studies have suggested that cancer-associated alterations in tumour cell biology including changes in post-translational modification (PTM) patterns may also lead to novel antigens that can be directly recognized by T cells. The PTM-derived antigens provide tumour-specific T-cell responses that both escape central tolerance and avoid the necessity for individualized therapies. PTM-specific CD4 T-cell responses have shown tumour therapy in murine models and highlight the importance of CD4 T cells as well as CD8 T cells in reversing the immunosuppressive tumour microenvironment. Understanding which cancer-specific antigens can be recognized by T cells and the way that immune tolerance and the tumour microenvironment shape immune responses to cancer is vital for the future development of cancer therapies.

## Graphical Abstract

**Graphical Abstract:**
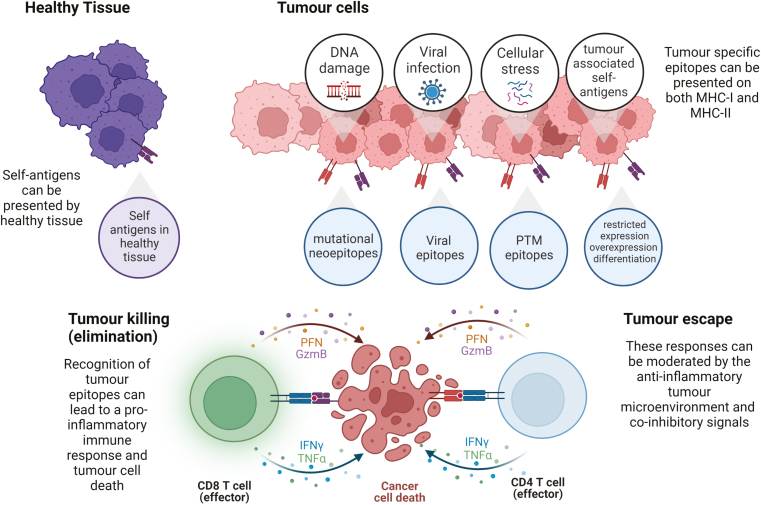

## Introduction

The interaction between the immune system and cancer is widely acknowledged in the literature. A dynamic cellular communication between tumours and immune cells including T cells can regulate tumour growth and progression. Inflammation is an essential process at different stages as it can both promote tumorigenesis and suppress tumour growth by altering the host immune response to the tumour [1]. This is known as the concept of ‘cancer immunoediting’ which postulated that the host’s immune system can inhibit cancer growth and promote cancer progression simultaneously. Cancer immunoediting consists of three different phases: elimination, equilibrium, and escape [2]. At the initial stage of tumorigenesis, immune cells respond to tumour-expressed antigens and produce inflammatory and potentially protective anti-tumour responses. This elimination phase starts when the innate and adaptive immune cells recognize pre-malignant lesions (newly transformed cells) and kill growing tumours, thus maintaining protection against cancer.

Apoptotic or necrotic tumour cells release intracellular molecules and antigens as well as damage-associated molecular patterns that recruit both adaptive and innate immune cells (dendritic cells [DCs], macrophages, CD4^+^ and CD8^+^ T cells, and natural killer [NK] cells). The production of IFNs, mainly IFN-γ, can mediate the recruitment of other innate immune cells to the site of the growing tumour whilst also inhibiting angiogenesis and growth of tumour cells [3]. Professional antigen-presenting cells (APCs), such as DCs, process and present tumour antigens in complex with major histocompatibility class I and II molecules (MHC-I or MHC-II) for the stimulation of CD8 and CD4 T cells through TCR-mediated recognition [4]. TCRs have the capacity to be cross-reactive and recognize multiple peptide/MHC complexes [5, 6] as such some TCR therapies have shown adverse clinical effects due to currently unpredictable cross-reactivity [7, 8]. However, to increase specificity, T-cell function is influenced by both the affinity of the single TCR-peptide/MHC engagement and combined multiple TCR-peptide/MHC interactions with co-receptor signals as a measure of avidity (reviewed in [9]). Circulating CD4 T cells are often the first to migrate to the tumour site. Upon encountering their cognate antigen either expressed on MHC class-II-positive tumour cells or on infiltrating APCs, Th1 CD4s release cytokines such as IL-2 that maintain the function and viability of CD8^+^ T cells and interferon gamma (IFNγ) and tumour necrosis factor alpha (TNFα) which can directly induce apoptosis of target cells and upregulate MHC molecules to enhance T-cell killing. Furthermore, alongside NK cells, they induce an inflammatory cascade to promote the extravasation of other immune effector cells such as CD8 T cells whose TCRs recognize peptide presented on MHC class I within the tumour environment. In addition, unconventional T cells such as mucosal-associated invariant T (MAIT) cells recognize non-peptide antigen in the context of MHC class-I-related protein MR1 (reviewed in [10]) and non-MHC-restricted gamma delta T cells [11] are detected in tumours. Evidence of infiltration into tumours is seen but these can be both tumour promoting as well as prevent tumour growth under different conditions [10, 11].

The contribution of tumour-specific adaptive immunity may vary and a major factor to consider is the heterogeneity within and between cancers. Studies using immunocompetent mouse models have demonstrated the importance of T cells in the rejection of tumours [12]. Mutations accrued by tumours during the transformation process can be efficiently targeted by T cells although those with low mutational burden are more difficult targets for T-cell therapy [13]. Both these observations highlight that T cells can distinguish between tumour and self. The ability of patients’ T cells to recognize cancer is complicated by the extensive HLA polymorphisms seen in humans which leads to differences in their capacity to present and recognize tumour-associated antigens. It is not only pro-inflammatory T cells that respond to antigens within the tumour microenvironment, regulatory T-cell subsets also respond to cognate antigens [14] to exert suppressive effects and promote tumour growth. Immune surveillance is dependent upon a variety of immune cell types and must be a continually ongoing process each time new antigenically distinct tumour cells develop [15].

This review will discuss the targets that T cells can ‘see’ within tumours and the hurdles they must overcome to maintain the elimination phase and tumour immune surveillance.

## Identification of cancer-specific T-cell targets

Many years have been spent developing techniques to identify the cancer-specific targets of T cells. In the 1960s, the concept that tumours could be recognized by the immune system was accepted but it wasn’t until 30 years later that the first tumour-specific antigens recognized by T cells were identified (reviewed in [16]). Early identification of antigens involved the establishment of cytotoxic T-cell (CTL) cultures and clones that specifically recognized tumours. This field further progressed after the discovery that T cells recognized peptide determinants presented on major histocompatibility complexes. Studies, first in mouse models and then in humans, examined T-cell clone recognition of an HLA-matched cell line transfected with gene libraries of a specific tumour model [17–19]. To further map the specific MHC-bound peptides recognized by the T cells transfectant studies were performed with short cDNA fragments rather than genomic libraries [20]. Methods then expanded to employing antibody responses from cancer patients to identify proteins present in cancers that could be targeted for T cells. A technique known as ‘serological analysis of recombinant cDNA expression libraries’ (SEREX) identified many of the well-known cancer testis antigens [21]. Once candidate antigens had been identified, the use of peptide libraries spanning the antigen sequences could also be used to narrow down the peptides to which T cells responded.

In the 1990s, Rammensee *et al*. pioneered an approach involving the elution of peptides from MHC molecules and peptide identification by mass spectrometry to identify potential T-cell targets [22, 23]. Despite its success, limitations of this method include the stability of peptides throughout the methods used, the low copy number of some peptides, and that some peptides are not detectable by mass spectrometry. In addition, the high cell numbers required for this technique mean that often tumour cell lines and not primary tumours have been used for sequencing which may not be representative of the primary tumour. Although technically demanding, this technique is the one that permits the identification of post-translational modifications (PTMs) [24, 25]. This revolutionary approach also enabled the establishment of epitope prediction algorithms which are currently widely used in reverse immunology approaches for target identification [26]. Predicted or identified peptides are tested for recognition by T cells *in vitro* although it is essential to assess the recognition of naturally processed peptides as many *in silico* predicted peptides may not be produced by the antigen processing machinery (reviewed in [27]).

More recently, whole exome sequencing and next-generation sequencing have enabled the identification of cancer-specific genome mutations that have the potential to generate neoepitopes. This often requires tumour tissue samples for isolation of genetic material; however, the discovery of circulating tumour DNA (ctDNA) in the bloodstream may provide the opportunity to genetically map the tumour without the need for a solid biopsy [28]. Identified candidates are then assessed using *in silico* prediction software and assessed for immunogenicity [29, 30]. This includes utilizing databases such as the immune epitope database (IEDB http://tools.iedb.org). Methods used include functional T-cell assays as well as multimer-based T-cell detection (reviewed in [30]).

In addition, methods such as interaction-dependent fucosyl-biotinylation [31], detection of activation markers [32], single-cell RNA and TCR analysis [33], yeast display [34], and CRISPR-Cas9 screening [35] have all been employed to isolate and characterize tumour-specific T cells. As technologies evolve, there will certainly be an expansion in the identification of potential T-cell targets that can be exploited for tumour immunotherapy.

## Tumour antigens recognized by T cells

### Tumour-associated antigens

The first antigens to be identified as TAAs were those over-expressed in tumours, associated with differentiation or expressed in malignant tissues with restricted normal expression such as cancer testis antigens. T-cell responses have been identified from regressing cancer patients to differentiation antigens such as tyrosinase-related protein (TRP) 2 and gp100 and the cancer testis antigens NY-ESO-1 and the MAGE family [36–38]. Cancer testis antigens include targets such as MAGE, GAGE, LAGE, and BAGE families and many others. More recently, our work has added the HAGE antigen to this list [39]. Many more antigens are now classified as cancer testis antigens that are potential targets for T cells in tumours (reviewed in [40]). Immunotherapies targeting differentiation antigens may be associated with increased toxicity due to wider expression on normal tissues (reviewed in [41]). In addition to gp100, TRP-1, and TRP-2, antigens in this category also include Melan A/MART-1, tyrosinase, prostate-specific antigen, and carcinoembryonic antigen (CEA). Many groups including ours have investigated targeting this group with immunotherapies and vaccines [42–45]. Our work with a DNA vaccine, SCIB1, has shown vaccine-mediated stimulation of responses to gp100 and TRP-2 antigens in melanoma patients. A T-cell response was detected in all 20 fully resected patients, and they remained alive with a median observation time of 37 months [43]. Over-expressed antigens recognized by T cells include candidates such as HER2, hTERT, MUC1, WT1, survivin, and many more. The low-level expression of these on normal tissues can be challenging for targeted therapies. Indeed, TCR-based therapies targeting cancer testis antigen MAGE-A3 and MAGE-A12 with high avidity have demonstrated their potential limitations with normal tissue toxicity, in particular in the brain, or cross-reactivity with a peptide expressed by cardiac cells [7, 8]. Preferentially Expressed Antigen in Melanoma (PRAME) is another cancer testis antigen that is being incorporated in to TCR-based immunotherapies [46]. This antigen is associated with poor prognosis and metastasis in uveal melanoma making it an attractive target for immunotherapies [47, 48]. There is also emerging evidence that these tumour-associated antigens can be targets for regulatory T-cell populations within tumours [14, 49–51]. Expression of these cancer antigens at lower levels on healthy tissue and their subjection to tolerance mechanisms may contribute to the development of a regulatory phenotype. This has led to some groups moving towards targeting other types of TAAs that are less likely to be the subject of immune tolerance.

### Virus-associated cancers and viral antigens

Viral infection can alter the cell cycle and increase the risk of developing tumours. The tumours attributed to viruses vary by cancer type and viral infection type [52]. Human papillomavirus (HPV) infection is linked to anogenital carcinomas, contributing to almost 100% of cervical carcinomas but only 4% of the oral cavity and laryngeal cancers (reviewed in [52]. Overall, oncoviruses have been estimated to be responsible for around 10% of human cancers worldwide [53]. Prophylactic vaccines that prevent viral infection can eliminate virus-induced cancers before they can develop. This early intervention strategy is particularly important in developing countries where the cost of immunotherapies is prohibitive. But importantly, in established oncovirus-induced cancers, cells have the potential to express viral antigens. These non-self, tumour-specific antigens can be presented via MHC and provide important targets for T cells and have not been subject to immune tolerance.

Oncogenic viral antigens have been identified in HPV-associated cervical cancer, hepatitis B virus (HBV)-associated hepatocellular carcinoma, and human herpesvirus-8-associated Kaposi sarcoma, as well as human T-cell leukaemia virus type 1 (HTLV-1). HBV is the leading cause of hepatocellular carcinoma worldwide, and trials have begun using T cells engineered to express HBV-specific TCRs to treat chemo-resistant extrahepatic metastases [54]. HPV-specific T cells infiltrating cervical cancer and draining lymph nodes have been shown to be restricted to the less common HLA-DQ and HLA-DP alleles [55]. EBV-specific T cells have been used against nasopharyngeal carcinoma, post-transplant lymphoproliferative disease (PTLD), Hodgkin’s, and non-Hodgkin’s lymphoma as summarized by Leung and Heslop [56] with evidence of clinical responses in all indications but particularly in the prevention of EBV-induced PTLD when disease burden is low. In HTLV-1, the TAX oncoprotein, which is critical to disease, has been shown to be a viable target for vaccines which induce multi-epitope T-cell responses [57].

Viral immune responses will specifically target tumours and virally infected, pre-malignant cells with minimal on-target, off-tumour effects suggesting a good safety profile. Vaccination against oncogenic E6 and E7 proteins from HPV-16 showed vaccine-induced T-cell responses and a benefit for women with pre-malignant vulvar intraepithelial neoplasia [58]. In HPV-16-associated cancers, therapeutic clinical efficacy was correlated with HPV-specific immune responses in patients treated with HPV peptide vaccine and checkpoint blockade [59, 60]. Due to the tumour-specific nature, these antigens are safe as targets for prophylactic vaccines and preventative vaccines also have obvious advantages for patients. The ideal for prophylactic vaccines is to induce immune responses that neutralize virus infection, therefore, preventing virus-directed oncogenic changes although not all infections lead to oncogenic transformation. Targets for prophylactic vaccination, therefore, may include antigens not necessarily expressed by malignant cells but those expressed by the virus upon infection. These approaches have been recently reviewed [61] and include prophylactic vaccines for HPV and HBV. Multiple HPV vaccines have shown high levels of protection in large-scale trials aimed at inducing prophylactic antibody responses [61–63] but the effectiveness of these HPV vaccines at inducing virus-specific T-cell responses for therapeutic cancer treatment relies on the stimulation of responses to viral oncoproteins and, therefore, may be limited by the relative contribution of the virus to cancer development. Some viruses such as HBV and EBV can remain latent and ‘invisible’ to the immune system for many years after infection resulting in challenges for immune-mediated targeting.

Unfortunately, viral antigens may fail to elicit good immune responses in a therapeutic setting. This may be due to the immunoregulatory tumour microenvironment or T-cell anergy due to chronic antigen exposure [64, 65]. HBV- and HCV-associated hepatocellular carcinoma is associated with a chronic viral infection that can lead to T-cell exhaustion and dysfunction [66]. Some researchers have argued that viral antigens may not be stably expressed, particularly in HBV-related HCC cells [67]. Viruses are also often adapted to avoid or subvert immune responses, and this can make it difficult to induce virus-specific immune responses. HBV and EBV both establish latency with a restricted protein expression profile. EBV and KSHV possess a number of immune subversion mechanisms which can prevent the presentation of viral antigens but also other self-antigens in virally infected cells (reviewed in [68]). HPV expresses a low level of viral proteins, does not cross the basement membrane, and does not cause cell death or protein secretion, thus reducing the presentation of viral antigens [69]. It does influence keratinocytes to depress innate immune responses and can affect T-cell immunity [70]. The HPV oncoprotein E7 has been shown in some studies to down-regulate CD8 T-cell responses leading to the failure of E7-immunized mice from controlling the tumour growth [71]. This has led to researchers using mutated E7 antigens with altered intracellular targeting to induce the effective immune responses [72]. In this case, additional therapies such as checkpoint inhibitors may be required to allow the virus-specific T cells to be effective. In patients, synergy has been shown between nivolumab and E6/E7-directed vaccination [59, 60]. In 2018, the FDA approved the anti-PD-1 antibody pembrolizumab for the treatment of recurrent or metastatic cervical cancer and the Checkmate 358 trial suggested no additional safety concerns were raised by this therapy [73]. Interestingly, treatment with checkpoint blockade has unmasked effector CD4 responses to cytomegalovirus (CMV) in melanoma patients suggesting that checkpoint blockade can expand responses to viral antigens [74].

### Mutated antigens and neoepitopes

Somatic mutations in cancer cells can generate neoepitopes that are recognized by autologous T cells. As these mutations are localized, the resulting neoepitopes are not subject to central tolerance and are also not found on healthy tissues making them appealing targets for cancer immunotherapy. The number of somatic mutations correlates with T-cell infiltration and patient survival across different tumour types [75]. The mutation rate is also predictive of cancer indications that will respond to checkpoint immunotherapy [76]. While some shared neoepitopes have been identified, the majority are highly individual and, therefore, are rarely shared between patients. The neoantigenome is the unique profile of neoepitopes and MHC–neoepitope interactions that are found in individual patients. Understanding complex neoantigenomes requires in-depth analysis that may lead to the development of individualized therapeutic vaccines [77]. The higher the mutational burden in the tumour, the easier it is to identify potential neoepitopes; however, the profile in the primary tumour does not always reflect the profile seen in metastasis. In humans and preclinical mouse models, mutational tumour neoepitopes have been demonstrated to be targets of T cells [78].

Lang *et al*. have categorized neoantigens into guarding neoantigens, restraining neoantigens, and ignored neoantigens. The extremely rare guarding neoantigens are drivers of early priming and rapid expansion of anti-tumour T cells. In contrast, restrained epitopes induce inactive immune responses due to the immunosuppressive effects of the tumour microenvironment. Finally, the most common form of neoepitope is the ignored neoepitopes that do not induce relevant immune responses alone but that can drive immunity when primed in the context of vaccines [29, 77]. This is not the only way to categorize neoepitopes, but it emphasizes that T-cell recognition of neoepitopes is not always sufficient to drive an effective immune response against cancer cells.

Neoepitopes can result from a number of mutations within the cells. One of the best studied is single-nucleotide variants in coding regions [79]. These mutations were the basis for the earliest clinical trials targeting neoepitopes [80, 81]. However, other cancer-specific mutations such as single insertions or deletions of nucleotides (indels), chromosomal rearrangements joining two unrelated fragments and alternative splice variants that lead to frameshifts may have more widespread impacts on coding regions and lead to the generation of multiple neopitopes [75, 82–84]. These mutations can generate high numbers of potential neoepitopes that could be recognized by T cells. While mutational burden can vary between cancers and individual patients, it is important to note that not all mutations will lead to neoepitopes that can induce anti-cancer immunity. Most mutations are not efficiently presented on MHC or recognized by T cells and are therefore not associated with neoepitopes. Computational systems have been developed to help identify which neoepitopes are likely to be presented in the context of specific MHC [30, 85]. But even among the mutations that can potentially generate immune responses, not all lead to tumour rejection [77, 86]. In addition, the identification and potential targeting of neoepitopes can also provide challenges when using peptide or protein products as not all neoepitopes can be easily synthesized. Despite this groups have successfully targeted mutational neoepitopes to stimulate tumour-specific immunity in patients. RNA-, peptide-, and DC-based vaccines have been trialled and shown enhancement or induction of responses with evidence of clinical benefit [80, 81, 87–89]. Interestingly, many responses detected when targeting longer epitope sequences were CD4 mediated which is reflected in murine preclinical models [29]. Studies on neoepitope responses have suggested in mouse models the requirement for both CD4- and CD8-mediated responses that act in cooperation to provide tumour therapy [90]. In addition to the initial RNA- and peptide-based vaccine trials, personal DNA-based vaccines encoding mutational neoepitopes and virus-vectored vaccines have been investigated with evidence of immunogenicity, partial responses, and stable disease [91]. These trials provide a rationale for the development of this approach in patients whose tumours have a high mutation frequency, particularly in combination with checkpoint blockade, and highlight the importance of CD4 T-cell recognition of tumours in combination with CD8 T-cell responses.

The heterogeneity seen within and between tumours may arise from epitope loss variants which would be time consuming and costly for individualized patient therapies. An alternative is to target ‘driver’ mutations that are relevant to a number of individuals rather than patient specific. These are being widely investigated in therapies for blood malignancies such as AML and CML. An example of chromosomal translocation is the BCR-ABL driver mutation, somatic driver mutations in p53 and Kras, and a frameshift mutation that seen in NPM1 (reviewed in [92]).

Mutational neoepitopes are promising immunotherapy candidates in tumours with a high mutational burden, but many tumours maintain a low mutational burden, and identification of T-cell targets within these tumours remains a priority.

### Post-translational modifications

In addition to the classical gene-derived neoepitopes, altered epitopes can be formed by post-translational splicing of peptide fragments from within or between proteins [91, 92]. Tumours can also display altered patterns of PTM on specific amino acids as a result of altered tumour cell biology or environmental influences (summarized in Table 1). PTM is an important regulatory mechanism that involves spontaneous or enzyme-mediated amino acid alteration. PTMs influence protein properties such as the charge state, conformation, hydrophobicity, and stability, ultimately affecting their function. PTMs can also impact protein processing, thereby influencing the repertoire of peptides presented on MHC molecules and diversifying the collection of naturally occurring peptides [93]. Modification of self-antigens can generate neoantigens that are not present at the time of thymic selection and possess the potential to be recognized by T cells in the periphery [94]. Various cellular stresses associated with disease and inflammation result in an increased level of reactive oxygen species (ROS) and enzymes that lead to modifications of amino acids.

**Table 1:** PTMs detected in oncogenesis

PTM	Amino acid modified	Example of protein(s) modified
Phosphorylation	Serine, threonine, tyrosine	EGRF, p38 MAPK, p53, STAT3, NF-κB, IRS2, CDC25B, BCAR3
Acetylation	Lysine	Histone
Glycosylation	Serine, threonine, asparagine	MUC1, MUC16. PD-L1,
Citrullination	Arginine	Histone, β-catenin, Vimentin, α Enolase, Nucleophosmin
Homocitrullination	Lysine	Aldolase, Cytokeratin 8, BiP(GRP78), Vimentin
Palmitoylation	Cysteine, serine, lysine, histidine	N-Ras
Ubiquitination	Lysine	A20, PTEN, Mdm2, 20S proteasome
SUMoylation	Lysine	HSPs, c-Myc, Ubc9
Nitration	Tyrosine	Histone deacetylase 2
Deamidation	Asparagine	BCL-xL
Methylation	Arginine, lysine, glutamic acid, aspartic acid, asparagine	P53, NF-κB, PI3K, ERα, PTEN

**Figure 1:**
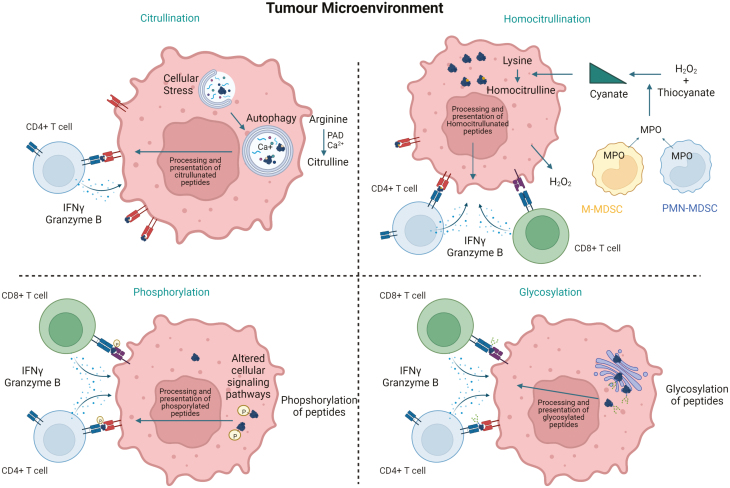
Schematic of post-translational modifications (PTMs) in tumour. Tumour cells are subject to conditions that can lead to altered patterns of PTM. PAD-dependent citrullination of arginine and MPO-dependent homocitrullination of lysine as well as altered patterns of phosphorylation and glycosylation can all occur in tumour cells. Cellular processing can then lead to presentation of these modified epitopes via MHC-I and in the presence of IFNγ, MHC-II. This allows CD4 and CD8 T cells to directly recognize tumour-associated PTMs.

Phosphorylation is the most common form of PTM that is often dysregulated in cancer cells leading to uncontrolled cell growth, differentiation, and metastasis (Figure 1). The binding affinity of phosphopeptides to HLA-A2 is stronger than the unmodified counterpart due to interactions of phosphorylated residues with HLA-A2. Additionally, the solvent-exposed hydrophilic property of phosphate suggests potential direct contact between phosphorylated moiety and the TCR receptor, hence resulting in increased peptide immunogenicity [95]. Phosphorylated peptides have been reported to be processed and presented on both MHC class I and II molecules eliciting CD8+ and CD4+ T-cell responses, respectively [96, 97]. Zarling *et al*. have described two phosphopeptides, one from insulin receptor substrate 2 and another from cell division cycle 25b (CDC25B) as CD8+ T-cell epitopes that are presented by multiple HLA-A2+ cancers [98]. Another HLA-A2-restricted phosphopeptide has been described from breast cancer anti-oestrogen resistance 3 (BCAR3) that induces responses capable of tumour regression in a preclinical model [96]. CD8 T cells recognizing phosphopeptides presented on tumour MHC class I have also been identified in colorectal cancer and leukaemia patients [99, 100]. First human clinical trials using CD8+ T-cell-specific phosphopeptides as vaccines in high-risk melanoma patients have demonstrated vaccine immunogenicity and safety but clinical responses are yet to be demonstrated [101].

Protein citrullination is well established as being associated with the pathogenesis of autoimmune diseases but it has also been detected in cancer [102, 103]. Citrullination is the conversion of positively charged arginine to neutrally charged citrulline (cit) in a Ca2+-dependent enzymatic process driven by peptidyl arginine deaminase (PAD) enzymes (Figure 1). Citrullination occurs during times of cellular stress when intracellular Ca^2+^ levels are elevated, and autophagy is induced to provide additional energy. Citrullination influences the ability to form hydrogen bonds, alters protein structure and cleavage, and can result in different peptides produced in stressed versus healthy cells [104]. In the presence of appropriate inflammatory cytokines, citrullinated peptides are presented on MHC class-II molecules which can lead to CD4+ T-cell activation [105, 106]. CD4 T-cell responses have been detected by our group and others in healthy humans and murine models that are restricted through a number of HLA alleles and not those just associated with autoimmune disease suggesting this may be a mechanism to detect cellular stress [107, 108]. Indeed, T cells specific for citrullinated peptides can be detected in healthy donors and ovarian cancer patients, thus implying that T-cell repertoires to citrullinated peptides are not subject to thymic deletion and tolerance [109–111]. Autoimmunity is rarely induced by T-cell responses alone and requires, tissue damage, cellular apoptosis, and release of citrullinated proteins to stimulate antibody responses for the formation of immune complexes that drive the disease. In tumours, autophagy is up-regulated as a pro-survival response, which in turn can result in increased citrullination [105]. The presence of increased levels of PAD enzymes and citrullinated proteins has been reported in breast cancer [103]. Our research has focused on targeting citrullinated epitopes expressed by tumour cells. Strong cit-specific CD4+ T-cell responses could be stimulated by peptide vaccination in HLA-transgenic mouse models [106, 109, 112]. These robust T-cell responses were associated with potent anti-tumour immunity in murine melanoma, lung, pancreatic, and ovarian tumour models. Responses specific to citrullinated vimentin and enolase were able to induce tumour regression in the aggressive murine B16 melanoma model within 4 days of a single vaccination [111]. Murine models demonstrated no sign of toxicity suggesting the presence of these modified peptides in cancer cells rather than healthy cells and that these could act as targets for tumour therapy [106, 109, 111, 112]. The targeting of citrullinated peptides for tumour therapy is currently being assessed in clinical studies (NCT05329532). Initial evidence also suggests that citrullinated self-proteins have the potential to be recognized by Th3 cells [113]. Indeed, mass spectrometry analysis of citrullinated peptides expressed on MHC class II molecules on murine B16 melanoma demonstrates that citrullinated self-peptides can stimulate regulatory T cells [25]. Perhaps suggesting that citrullinated peptide-specific repertoires can be polarized and perhaps could be harnessed as a mechanism to prevent autoimmunity.

Another example of a PTM that is well documented in the context of autoimmune disease but less so in cancer is homocitrullination. Unlike citrullination, homocitrullination is a non-enzymatic process involving the chemical reaction between cyanate or its active form isocyanic acid and the amine (NH_2_) groups of lysine forming homocitrulline (Hcit) (Figure 1). Increase in cyanate can result from the breakdown of urea that occurs in conditions of renal failure [114]. Inflammation can also enhance homocitrullination via the action of myeloperoxidase enzyme (MPO) enzyme predominantly released by neutrophils [115]. However, tumour-associated myeloid-derived suppressor cells (MDSCs) are also a source of MPO driving homocitrullination in the tumour microenvironment [116, 117]. We have identified homocitrullinated peptides from proteins expressed in many solid cancers such as vimentin, aldolase, cytokeratin 8, enolase, and binding immunoglobulin protein (Bip) that are recognized by CD4+ T cells [116, 118]. Vaccination of HLA-transgenic mice with homocitrullinated peptides stimulates potent anti-tumour immunity that is CD4 T cell dependent and provides survival benefits with no related toxicity [116, 118]. In preclinical mouse models, our recent unpublished data demonstrate the detection of CD8+ T-cell responses to homocitrullinated peptides that can provide tumour therapy against the aggressive murine B16 melanoma model suggesting that homocitrullinated peptides are also recognized by CD8 T cells in the tumour microenvironment. T cells specific for homocitrullinated peptides can also be detected in healthy donors implying these specific T-cell populations can escape central tolerance [116, 118]. Our preclinical data suggest that citrullinated and homocitrullinated peptides are excellent vaccine targets and can be harnessed in the development of future cancer vaccines [109, 111, 116, 118].

In addition to citrullination, homocitrullination, and phosphorylation, other PTMs such as glycosylation, isomerization of aspartate, deamidation of glutamine, and nitration of tyrosine are known to be recognized by T cells but their role in T-cell surveillance of cancer remains to be determined. Glycosylation is a PTM known to be up-regulated in cancer and can be a specific target of antibodies (Figure 1). Glycopeptides have been detected bound to MHC class I and II molecules (reviewed in [119]) and have been shown to be neoantigens recognized by T cells in leukaemia [120]. One example of an O-glycosylated protein that is being targeted for CD8 T-cell-mediated cancer therapy is MUC1 [121]. The isomerization of aspartate residues can also generate altered T-cell epitopes [122] although a role for this modification in cancer is yet to be determined. Two other PTMs that are known to stimulate new modification-specific T-cell responses are deamidation of glutamine and nitration of tyrosine or tryptophan [119]. Deamidation of glutamine is mediated by the enzyme tissue transglutaminase 2 which can be present in tumours [123], and therefore, there is potential for this modification to be a target for T-cell recognition in tumours. Nitration of tyrosine or tryptophan occurs in the presence of ROS such as nitric oxide (NO) released by activated macrophages and DCs. Given the presence of ROS in tumours, this modification also has the potential to be a target for T cells in cancer, but this remains to be determined.

### Alternate non-MHC-restricted T-cell targets

Unconventional T cells such as MAIT cells, MR1 restricted cells and gamma delta T cells recognize targets in a non-MHC-restricted manner. Gamma delta T cells can respond to stress-induced molecules on tumour cells such as MHC class I polypeptide-related sequence A (MICA). This is often up-regulated in cancer cells compared to normal cells and target cells are recognized through NKG2D association and/or the gamma delta TCR [11] although little is yet known about the targets of the gamma delta TCR. Invariant T cells such as MAIT cells recognize targets via the MHC class-I-related protein MR1 that can be up-regulated on cells under metabolic stress and has been detected in tumours [10, 124]. Recognition is of small metabolites such as vitamin B metabolites and riboflavin synthesis intermediates associated with MRI molecules but MR1 molecules may also permit the association of bacterial antigens and tumour antigens [124]. A population of MR1-restricted T cells has been identified that respond to an unidentified cancer metabolite on cancer cells [35].

## Influence of the tumour microenvironment on tumour infiltrating T cells

Despite the ability of T cells to recognize specific tumour antigens within the tumour microenvironment, there remain barriers to the infiltration of T cells into tumours. Tumours can be classified into immune-rich or ‘hot’ tumours, and these are often associated with high tumour mutational burden [125]. However, many tumours are classified as immune poor or ‘cold’, desert and excluded tumours where there is little or no immune infiltrate [125]. Even if T cells infiltrate into tumours, they can also be affected by the cytokine milieu and co-stimulatory molecules that can either repress or enhance signals received through the TCR (summarized in Table 2). Co-stimulatory molecules such as toll-like receptor [126], CD28, ICOS, and OX40 enhance T-cell activation whereas negative mechanisms through molecules such as CTLA-4, LAG-3, TIM-3, and PD-1 pathways lead to the repression of T cells, anergy, and exhaustion (reviewed in [127]). Subversion of responses by tumour cells establishes a persistent inflammatory microenvironment (low-grade chronic inflammation) which further promotes tumour development [1].

**Table 2:** T-cell co-stimulatory and inhibitory molecules in the tumour microenvironment

T-cell stimulatory receptor	Ligand	Ligand expression
CD28	CD80 (B7-1) CD86 (B7-2)	B cells, activated T cells, DCs, macrophages, monocytes, some tumour cells
ICOS (CD278)	ICOS-L (CD275/B7-H2)	B cells, DCs, endothelial cells, macrophages, Monocytes
OX40 (CD134) *	OX40-L (CD252)	Activated T cells, Tregs, NK cells, endothelial cells, APCs
CD27	CD70	Activated DC, B cells and T cells, Lymphoid malignancies, some solid malignancies
4-1BB (CD137)	4-1BBL (CD137 ligand)	DC, B cells, macrophages, monocytes, various tumour cells
CD40L (CD154)	CD40	DC, B cells, macrophages, monocytes, endothelial cells, tumour cells
CD270	CD258, CD272, CD160	DC, activated T and B cells, NK cells, monocytes, macrophages, some tumour cells
GITR	GITR ligand	DC, B cells, macrophages, monocytes, endothelial cells, APCs, tumour cells
CD226 (DNAM-1)	CD112, CD155	APCs, tumour cells, epithelial cells, TAMs, monocytes
T cell Inhibitory receptor	Ligand	Ligand expression
CTLA-4 (CD152)	CD80 (B7-1) CD86 (B7-2)	B cells, DCs, activated T cells, macrophages, monocytes
LAG-3 (CD223)	MHC-II	T cells, B cells, DCs, macrophages, monocytes, tumour cells
TIM-3	Galectin 9	T cells, Tregs, many cell types
PD-1 (CD279)	PD-L1 (CD274/B7-H1) PD-L2 (CD273)	DCs, B cells, T cells, NK cells, macrophages, monocytes
CD160	CD270, MHCI	Resting T cells, B cell subsets, immature DCs, Tregs, monocytes, NK cells, some tumour cells Most cells
CD272 (BTLA)	CD270	Resting T cells, B cell subsets, immature DCs, Tregs, monocytes, NK cells, some tumour cells
PD-L1 (CD274)	CD80 (B7-1)	B cells, activated T cells, DCs, macrophages, monocytes, some tumour cells
CD112R (PVRIG)	CD112	APCs and tumour cells, epithelial cells, TAMs, monocytes
CD96	CD155	APCs, monocytes, tumour cells, TAMs
TIGIT	CD155, CD112, CD113	APCs, tumour cells, epithelial cells, TAMs, monocytes

*OX40 is inhibitory for Tregs.

DCs: dendritic cells, NK: natural killer cells, NKT: natural killer T cells, Th2: T helper 2 cells, Tregs: T regulatory cells, MHC-II: major histocompatibility complex class II, ICOS: inducible T-cell co-stimulator, LAG-3: lymphocyte activation gene 3, CTLA-4: cytotoxic T-lymphocyte-associated antigen 4, TIM-3: T-cell immunoglobulin and mucin domain 3, PD-1: programmed cell death protein 1, PD-L1: programmed cell death ligand 1, TIGIT: T-cell immunoglobulin and ITIM domain, GITR: glucocorticoid-induced TNFR-related protein.

The persistent low-grade inflammation in the absence of co-stimulation can result in T-cell anergy and the preferential stimulation of regulatory T cells (Tregs). CD4+ Tregs play a vital role in maintaining immune homeostasis and peripheral tolerance, thereby preventing autoimmunity. In cancer, Tregs are found enriched in the tumour microenvironment and mediate mechanisms that contribute to the inhibition of anti-tumour response and tumour progression [128]. Tregs can respond to specific tumour antigens [25, 49–51]. Upon recruitment and activation in the tumour microenvironment, Tregs exert their immunosuppressive activities via various mechanisms including (i) secretion of soluble immunosuppressive cytokines such as transforming growth factor beta (TGF-β) [129], interleukin-10 (IL-10), and interleukin-35 (IL-35), (ii) modulation of DCs, (iii) metabolic disruption, and (iv) suppression by direct cytolysis [130, 131].

Tumour infiltrating immune cells including T cells can also be affected by cancer-associated fibroblasts that can promote tumour growth and prevent and polarize T-cell function through the release of cytokines and chemokines such as TGFβ, IL6, indoleamine 2,3-dioxygenase (IDO), and CXCL12 (reviewed in [132]). In addition, MDSCs contribute to the immunosuppressive environment by promoting immune tolerance and releasing anti-inflammatory cytokines ROS and NO [133].

Evidence that anti-tumour T cells are affected by suppressive mechanisms mentioned above is highlighted through studies examining pathway blockade and depletion. These suppressive mechanisms can be overcome in certain circumstances through the blockade of inhibitory checkpoint molecules such as CTLA-4, the PD-1 pathway, and LAG-3 [134, 135], the depletion of myeloid suppressor cells [136] and the reversal of effects of suppressive cytokines and metabolic enzymes such as TGF-β [137], IL-10 [138], and IDO [139]. Thus, in addition to T-cell recognition of cancer antigens, there is a wealth of other molecules that T cells encounter that influence their function within the tumour microenvironment.

## Conclusion

In cancer patients, the natural T-cell response has been insufficient to clear the tumour and immunotherapy focuses on the re-education or stimulation of pro-inflammatory T cells with minimal on-target off-tumour reactivity. Understanding the types of antigens that can be recognized by T cells is vital to furthering this aim.

Cancer antigens can be derived from normal proteins that are expressed in tumours, virus-associated proteins, or epitopes that are altered due to cancer-associated mutation or PTMs. The latter has the advantage of exploiting natural tumour processes, being able to target proteins present in most tumours and thus applicable to patients across tumour types. New technologies are improving our ability to identify T-cell targets in patient and tumour-specific ways. However, the challenge is to induce responses in the context of the immunoregulatory tumour microenvironment. Tumour heterogeneity is important and factors such as the type of malignancy, the tumour mutational burden, and whether tumours are immune rich or immune poor/ excluded will play a role in the types of intervention that can be successful.

The success of checkpoint inhibitors in the clinic has demonstrated that mounting a strong pro-inflammatory T-cell-mediated immune response can lead to tumour regression [140–142]. By better understanding the tumour antigens that are recognized by T cells, it is hoped that future therapies can build on the success of checkpoint inhibitors to target tumours that remain unresponsive to current interventions.

## Data Availability

Not applicable.

## Abbreviations

AML: acute myeloid leukaemia
CML: chronic myeloid leukaemia
CMV: Cytomegalovirus
CTL: cytotoxic T lymphocyte
CTLA-4: cytotoxic T-lymphocyte antigen 4
CXCL12: C-X-C Motif Chemokine Ligand 12
DAMPs: damage-associated molecular patterns
DC: dendritic cell
DNA: deoxyribose nucleic acid
EBV: Epstein–Barr virus
FDA: Food and Drug Administration
HBV: hepatitis B virus
HCC: hepatocellular carcinoma
HCV: hepatitis C virus
HLA: human leukocyte antigen
HPV: : human papillomavirus
HTLV-1: human T-cell leukaemia virus type 1
ICOS: inducible co-stimulatory molecule
IDO: indoleamine 2,3-dioxygenase
IFN: interferon
IL: interleukin
KSHV: Kaposi sarcoma virus
LAG-3: Lymphocyte Activation Gene 3
MAIT: mucosal-associated invariant T
MDSC: myeloid-derived suppressor cell
MHC: major histocompatibility class
MPO: myeloperoxidase
MR1: major histocompatibility complex, class I-related
NK: Natural killer
NKG2D: natural killer group 2D
NPM1: nucleophosmin 1
PAD: peptidyl arginine deaminase
PD-1: programmed cell death protein 1
PTLD: post-transplant lymphoproliferative disease
PTM: post-translational modification
RNA: ribose nucleic acid
TAA: tumour-associated antigen
TCR: T-cell receptor
TGF-β: transforming growth factor beta
Th: T helper
TIM-3: T-cell immunoglobulin and mucin domain-containing protein 3
TNF: tumour necrosis factor
Treg: regulatory T cell
